# Parkinson’s disease: no NOP, new hope

**DOI:** 10.18632/oncotarget.13710

**Published:** 2016-11-29

**Authors:** Ludovico Arcuri, Daniela Mercatelli, Michele Morari

**Affiliations:** Department of Medical Sciences, Section of Pharmacology, and National Institute of Neuroscience, University of Ferrara, Ferrara, Italy

**Keywords:** alpha-synuclein, MPTP, nociceptin/orphanin FQ, Parkinson’s disease, SB-612111, Neuroscience

Nociceptin/orphanin FQ (henceforth N/OFQ) is the endogenous ligand of the so-called nociceptin opioid peptide (NOP) receptor, a.k.a. Opioid Receptor Like 1 (ORL1) receptor, a G-protein coupled receptor which couples to Gi/o and intracellular kinases, among which mitogen-activated protein (MAP) kinases [1].

In 2004, we provided the first evidence that N/OFQ might play a role in Parkinson’s disease (PD), showing that a peptide NOP receptor selective antagonist injected into the rat substantia nigra reticulata (SNr) reversed haloperidol-induced catalepsy [2]. This symptomatic effect was later replicated in various PD models, including 6-hydroxydopamine hemilesioned rats and 1-methyl-4- phenyl-1,2,5,6-tetrahydropyridine (MPTP)-treated mice or macaques [1], using potent, brain-penetrant small molecule NOP receptor selective antagonists, such as SB-612111 [3]. We also established that NOP receptor antagonists act into the SNr to rebalance GABA and glutamate inputs regulating the nigro-thalamic output pathway [4]. Indeed, endogenous N/OFQ tonically inhibits motor behavior [1], and nigral N/OFQ tone rises as a consequence of degeneration or impairment of nigro-striatal DA neurons, thus increasing motor constraint [1]. Besides sustaining motor symptoms, endogenous N/OFQ might also play a role in the neurodegeneration associated with PD. In fact, nigral DA neurons express the NOP receptor, and work from BM Cox lab revealed that genetic deletion of the pre-proN/OFQ gene confers mice partial protection against the neurotoxic effects of MPTP [5]. However, these experiments cannot unequivocally prove the involvement of endogenous N/OFQ in PD-like neurodegeneration since the ppN/OFQ gene codes for various biologically-active neuropeptides.

We recently addressed this issue, adopting a combined genetic and pharmacological approach [6]. First, consistent with data in ppN/OFQ^-/-^ mice, we found that acute MPTP (4 x 20 mg/Kg i.p., every 90 min) caused a lesser reduction of nigral DA cells in NOP receptor knock-out (NOP^-/-^) mice (40%) with respect to NOP+/+ controls (75%). This difference was physiologically relevant since MPTP-treated NOP^-/-^ mice were less akinetic/bradykinetic than MPTP-treated controls. Second, we showed that NOP receptor blockade with SB-612111 counteracted the MPTP-induced neurodegeneration in mice. Here, we administered MPTP subacutely (25 mg/Kg, i.p., once a day for 7 days) in order to induce a more gradual process of neurodegeneration and allow a clinically-driven protocol of SB-612111 administration. Indeed, PD is diagnosed when motor symptoms appear, i.e. long after the neurodegeneration has started. Thus, a disease-modifying agent not only should protect but also rescue dopamine (DA) neurons. As expected, mice subacutely treated with MPTP showed a mild reduction of nigral DA cells (27%) and striatal DA terminals (55%), and no motor deficits, due to functional compensation. SB-612111, given at 10 mg/Kg i.p., twice a day for 10 days, from the 4th day of MPTP administration onward, fully prevented the MPTP-induced neurodegeneration. To more convincingly prove the neuroprotective potential of NOP receptor antagonists, we finally adopted a more progressive model of PD-like neurodegeneration, in a different animal species: the rat overexpressing human mutated p.A53T α-synuclein (hα-syn), delivered into SNc through a recombinant adenoassociated viral (AAV) vector pseudotype 2/9 (AAV2/9 hα-syn). In this model, systemic SB-612111 administration (1 mg/Kg i.p., twice daily for 8 weeks, starting from one week after AAV2/9 hα-syn injection), conferred partial neuroprotection. Indeed, at the end of the 2-month treatment the number of nigral DA cells spared was significantly greater in SB-612111- treated rats (50%) than in saline-treated controls (25%). Considering that about 50% of nigral DA cells die a week after AAV2/9 hα-syn injection, i.e. at the time when SB- 612111 administration is commenced, this is a remarkable result. The protective effect was also observed at striatal DA terminals and, albeit partial, was enough to prevent the motor deterioration associated with the progression of neurodegeneration.

Light still needs to be shed over the possible mechanisms through which N/OFQ causes neurotoxicity, and to the translational potential of these findings. Data obtained in SH-SY5Y cells and primary DA neurons provide clues to the first answer, showing that N/OFQ can cause cell death through a NOP-sensitive, p38-MAPK-mediated pathway [7]. Instead, whether N/OFQ plays a role in human PD remains a matter for a conjecture. N/ OFQ and the NOP receptor are expressed in the human basal ganglia, and, in keeping with rodent data, we found that N/OFQ levels are 3-fold elevated in the CSF of PD patients compared to non-parkinsonian controls [8]. Moreover, postmortem analysis revealed a downregulation of ppN/OFQ gene expression in the SN of PD patients, which was viewed as a compensatory mechanism to prevent excessive NOP receptor activation [7].

In conclusion, we feel that data obtained through a clinically-driven protocol in two different PD models (neurotoxic and etiologic) and animal species (mouse and rat), convey a solid proof-of-concept that NOP receptor antagonists protect/rescue DA neurons from PD-like neurodegeneration. Although more evidence needs to be collected to claim that endogenous N/OFQ contributes to DA cell loss also in human PD, NOP receptor antagonists hold promise as symptomatic and disease-modifying agents in PD therapy [5, 6].
